# Reappraising Schwannoma-Hemangioma Composite Tumors as Synchronous Tumorigenic Entities With Conjoined Histomorphology: A Case Report

**DOI:** 10.7759/cureus.20724

**Published:** 2021-12-26

**Authors:** Subramaniam Ramkumar

**Affiliations:** 1 Pathology, Woodland Hospital, Shillong, IND

**Keywords:** scalp, peripheral nervous system neoplasm, cavernous hemangioma, tumor, hamartin, tuberin, schwannoma, co-occurrence, genetic etiology

## Abstract

The most common peripheral nerve sheath and vascular tumors are schwannomas and hemangiomas, respectively. These tumors can affect any organ system and usually occur as separate morphologic and diagnostic entities. Herein, we describe the case of a 24-year-old woman with a tumor demonstrating composite differentiation to both cavernous hemangioma and schwannoma in the scalp. The patient had a slow-growing subcutaneous scalp tumor in the occipital region with an insidious onset and progression. The patient underwent wide local excision for treatment and based on follow-up evaluations has remained asymptomatic with no signs of recurrence.

## Introduction

Synchronous development with cavernous malformation and nerve sheath tumor coexistence can occur in the same lesion as a rare entity. Based on previously documented studies, they can be characterized into two distinct types. The first type is known as a conjoined association wherein the cavernous malformation and nerve sheath tumor coexist as a single lesion. The cavernous malformation and tumor occur at separate locations in the second variant which is known as a discreet association [1-2]. Such former cases have been previously rarely documented. This study presents a classic case of conjoined schwannoma-hemangioma and the histomorphologic approach to diagnosis. Moreover, we discuss the tumorigenic molecular pathways involved in these tumors [3].

## Case presentation

**Figure 1:**
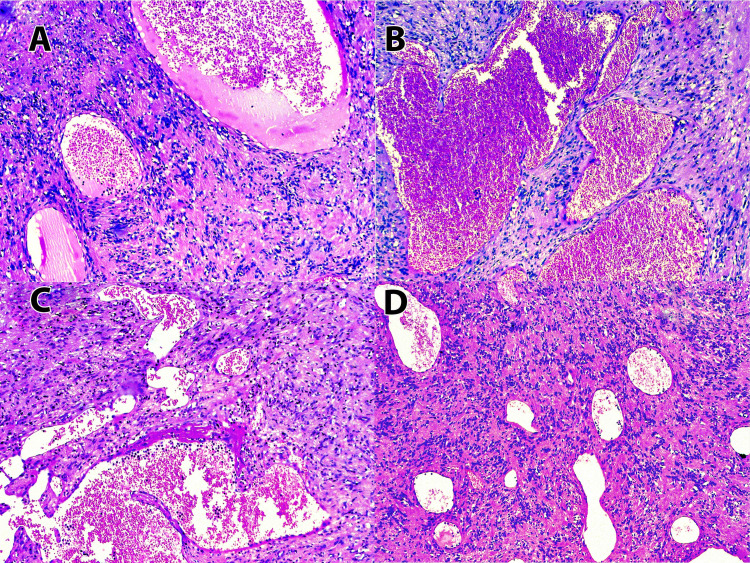
Tumor showing composite differentiation to schwannoma and hemangioma (H&E 20x).

A 24-year-old female patient presented to the surgical outpatient department with an insidious onset of swelling in the occipital region of the scalp. The plane of swelling was subcutaneous and measured 6 cm × 4 cm × 4 cm. The mass was firm and immobile. MRI of the swelling was performed with the following sequence: T1, T2, axial, Cor Flair, and PD Sag. A well-defined thin-walled lesion measuring approximately 4.5 cm × 4.3 cm × 3.3 cm was observed on the right side beneath the occipital bone. The lesion was observed in the subcutaneous plane abutting the muscle. It showed a heterogeneous signal on T2-weighted imaging with cystic changes and heterogeneous enhancement in postcontrast study. No abnormalities of the intracranial structures were noted. A clinical diagnosis of neurofibroma was made and a wide local excision was performed. Grossly, the surgical specimen consisted of skin with a subcutaneous mass together measuring 6.5 cm × 3.5 cm × 1 cm. The mass alone measured 4.5 cm × 4 cm × 3 cm. The cut surface of the mass was grayish white with numerous interspersed large, ecstatic, hemorrhagic, and cystic areas. Microscopic examination of the hematoxylin-eosin (H&E) stained sections showed the histomorphology of a composite tumor showing biphasic differentiation to schwannoma and cavernous hemangioma (Figure 1A-D).

**Figure 2:**
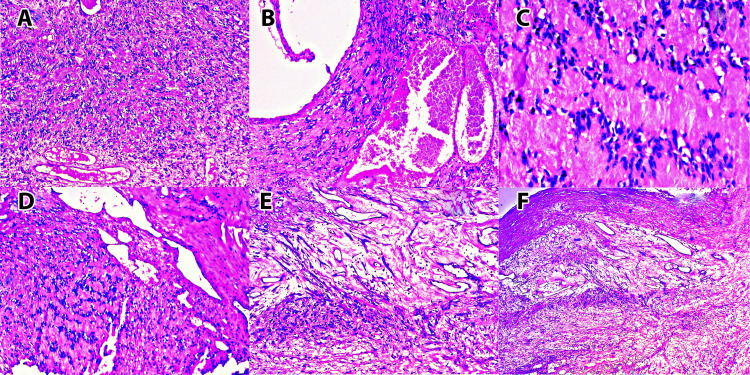
Tumor showing composite differentiation to schwannoma and hemangioma [A, B, D, E, F (H&E 20x), C (H&E 40x)].

The schwannoma component showed tumor architecture in the form of interlacing bundles, nests, and fascicles of cells. The cells were spindle-shaped, elongated, and wavy with tapered ends. They had spindle-shaped wavy nuclei, eosinophilic fibrillary cytoplasm, and indistinct cytoplasmic borders. Biphasic compact areas of hypercellular (Antoni A) and hypocellular (Antoni B) areas were noted. The cytoplasmic fibrillary processes of the cells showed prominent palisading of the nuclei around them (Verocay bodies) (Figure 2A-D). The hypocellular areas showed myxohyaline degeneration. Degenerative nuclear atypia was observed. Occasional mitotic figures were noted. Multifocal myxohyalinized areas and reticulocystic areas were also observed (Figure 2E-F).

**Figure 3:**
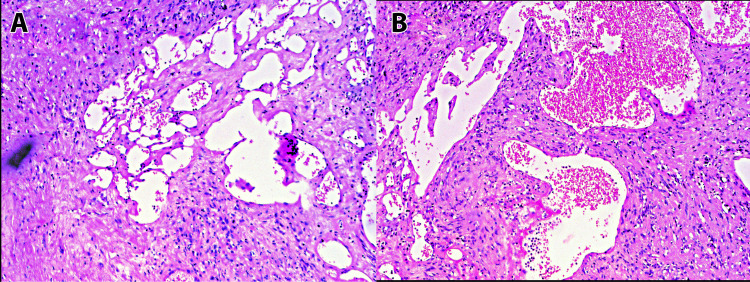
Hemangiomatous component showing lobular and plexiform proliferation of variably ectatically dilated vascular channels (A,B – H&E 20x).

The cavernous hemangioma component was interspersed throughout the hemangiomatous component. The hemangiomatous component showed numerous lobular proliferations of ecstatically dilated vascular channels (Figure 3A,B). The vascular channels showed luminal congestion and were lined by attenuated endothelial cells. Plexiform vascular channels and large ecstatic vascular channels with intraluminal pseudopolypoid projections were observed (Figure 3A,B). The stroma surrounding the vascular channels was fibrotic. The lesion partly adjoined the plane of excision.

**Figure 4:**
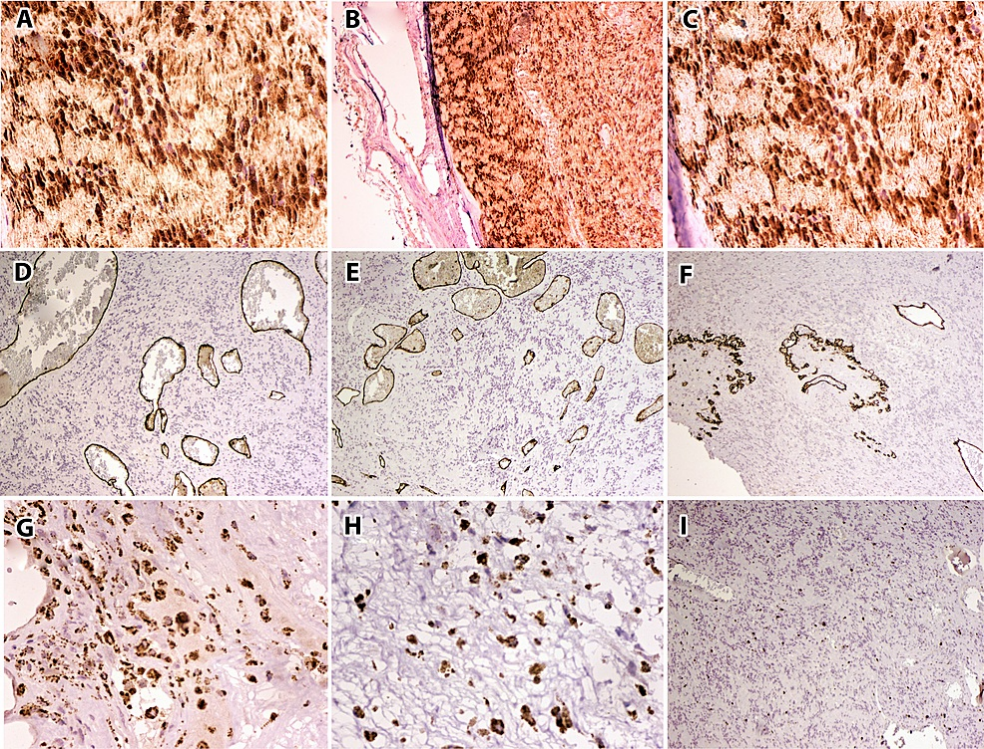
Immunohistochemistry of composite schwannoma-hemangioma. Immunohistochemistry showing schwannoma component positive for S-100 [A (40x) and B(20x)], calretinin [C (40x)]. The hemangiomatous component positive for CD34 [D,E, and F (20x)] and smooth muscle actin (SMA). CD68 positivity highlights numerous aggregates of macrophages throughout the neoplasm [G, H (20x)]. Ki-67 mitotic index in the schwannoma component was approximately 20%–30%, and in the hemangiomatous component around 10%–20% [I (20x)].

The studied sections of the lesion were negative for a definitive evidence of atypia or malignancy. Immunohistochemistry revealed that the schwannoma component was positive for S100 (Figure 4A [×40], B [×20]), calretinin (C [×40]) (Table 1), and epithelial membrane antigen, and was negative for PAN-CK, HMB-45, H-caldesmon, myogenin, desmin, factor XIIIa, and smooth muscle actin (SMA) (Table 1). The hemangiomatous component was positive for CD34 (Figure 4D-F [×20]) and SMA (Table 1). It was strongly positive for CD68, highlighting numerous macrophage aggregates throughout the neoplasm (Figure 4G,H [×20]) (Table 1). The Ki-67 mitotic indices in the schwannoma and hemangiomatous components were approximately 20%-30% and 10%-20%, respectively [Figure 4I (x20)] (Table 1).

**Table 1:** Details of antibodies used in the study. SMA, smooth muscle actin

Name of antibody	Source	Clone	Antibody dilution	Name of the supplier
HMB-45	Mouse monoclonal	HBM45	1:50 -1:100	PathnSitu, Livermore, CA, USA
Desmin	Mouse monoclonal	GM007	1:50-1:100	PathnSitu, Livermore, CA, USA
Caldemson	Rabbit monoclonal	EP19	1:50-1:100	PathnSitu, Livermore, CA, USA
CD34	Mouse monoclonal	Q. Bend.10	1:50-1:100	PathnSitu, Livermore, CA, USA
Ki-67	Mouse monoclonal	MIB-1	1:100	PathnSitu, Livermore, CA, USA
PAN-CK	Mouse monoclonal	AE1/AE3	1:50-1:100	PathnSitu, Livermore, CA, USA
EMA	Mouse monoclonal	E29	1:50-1:100	PathnSitu, Livermore, CA, USA
Calretinin	Rabbit polyclonal	Polyclonal	1:50-1:100	PathnSitu, Livermore, CA, USA
CD68	Mouse monoclonal	KP1	1:50-1:100	PathnSitu, Livermore, CA, USA
Myogenin	Mouse monoclonal	MGNI85+F5D	1:50-1:100	PathnSitu, Livermore, CA, USA
S-100	Rabbit monoclonal	EP32	1:50-1:100	PathnSitu, Livermore, CA, USA
SMA	Rabbit monoclonal	E184	1:50-1:100	PathnSitu, Livermore, CA, USA
Factor XIII-A	Mouse monoclonal	AC-1A1	1:100	PathnSitu, Livermore, CA, USA

Based on the above features, the benign composite tumor that developed showed divergent differentiation to schwannoma and cavernous hemangioma. The patient has been under follow-up with no recurrence or complications till date.

## Discussion

Peripheral nerve sheath tumors (PNSTs) are most commonly found in the upper extremities. Schwannoma, which is one of the most common PNSTs, affects the upper extremities and involves both sensory and motor nerves. Schwannoma is an encapsulated tumor that shows Antoni A and Antoni B regions on histology. Antoni A region is composed of spindle-shaped Schwann cells and is cellular. Nuclear palisading and Verocay bodies are frequently observed in cells within the Antoni A region. Antoni B region is also composed of Schwann cells, but their cytoplasm is hidden and their nuclei are surrounded by a thick myxoid matrix. Schwannomas show high levels of S100 on immunohistochemical analysis. If benign PNSTs of the extremities are stable in size and asymptomatic, they are treated conservatively. Excision is the surgical technique employed for the treatment of benign PNSTs. Because schwannomas in the extremities are encapsulated, they can be treated with enucleation or intraneural dissection, thereby saving the accompanying nerve [2].

The most frequently diagnosed soft tissue tumor of infancy and childhood is hemangioma, which affects 12% of all babies. They are more prevalent in females, Caucasians preterm children, twins, and mothers with more advanced maternal age [4]. Hemangiomas most commonly occur in the area of the neck and head (60%), trunk (25%), and extremities (15%). Based on the size of the vascular channels, hemangiomas are classified as cavernous large diameter hemangiomas or capillary small-diameter vascular haemangiomas. Infantile and congenital hemangiomas are two types of hemangiomas that are classified based on their growth.

Weakly constricted and abnormally dilated blood vessels are called cavernous hemangiomas, which have a flat endothelium lining. Categorization is based on distinct histological characteristics. The symptomaticity of cavernous hemangiomas is dependent on the tumor’s location. Larger lesions (>5 cm) are more likely to be symptomatic and vulnerable to consequences, including bleeding and rupture [4].

A total of 22 cases (including the present case) has documented the synchronous and conjoined occurrence of schwannomas and hemangiomas (Table 2).

**Table 2:** Cases of conjoined association.

Study	Year	Cases	Tumor type	Location of tumor
Sethi et al. [2]; Wilson [5]	2020 2016	1 1	Conjoined schwannoma/ vascular lesion	Left arm left temporal lobe
Feiz-Erfan [1]	2006	1	Conjoined schwannoma/ vascular lesion	Cranial nerve VIII
Asari et al. [6]	1992	1	Conjoined schwannoma/ vascular lesion	Cranial nerve V
Kasantikul et al. [7]	1987	1	Conjoined schwannoma/ vascular lesion	Parasellar
Kasantikul et al. [8]	1984	1	Conjoined schwannoma/ vascular lesion	Parapharyngeal
Kasantikul et al. [9]	1982	1	Conjoined schwannoma/ vascular lesion	Cranial nerve V
Pasquier et al. [10]	1980	1	Conjoined schwannoma/ vascular lesion	Mediastinum
Kasantikul and Netsky [11]	1979	7	Conjoined schwannoma/ vascular lesion	Cranial nerve VIII (4), spinal cord C1-2, peroneal nerve, ulnar nerve
Bojsen-Moller and Spaun [12]	1978	5	Conjoined schwannoma/ vascular lesion	Intraspinal (3), cranial nerve VIII, brachial plexus
Willis [13]	1967	1	Conjoined schwannoma/ vascular lesion	Posterior mediastinum

cases and intracerebral in one case. Seven cases were included in the biggest case series documenting the conjoined tumor relationship [1]. Based on the history, it is possible that our case is the 22nd case with the tumor occurring in the peripheral nerves. Based on the literature, the most prevalent concomitant relationship with cavernous malformations is PNST.In 8 and 10 cases, the tumors were present in the cranial and peripheral nerves, respectively. Furthermore, the tumor was intracranial in two

A shared molecular mechanism has been proposed to be involved in the development of these tumors. The common cell signaling pathways implicated in concomitant schwannoma/hemangioma tumorigenesis are the mitogen activated protein kinase (MAP kinase) pathway, the phosphatidyl inositol 3 kinase/mammalian target of rapamycin (PI3K/mTOR) signaling, and the phospholipase second messenger system pathways. Angiopoietin-tie 2 interactions and schwannoma-secreted vascular endothelial growth factor (VEGF) act as the preliminary mechanisms in initiating and maintaining the above-described complex gesticulating reactions between Schwann and endothelial cells, resulting in the simultaneous growth of schwannoma and hemangioma in the same tumor [3].

Furthermore, certain researchers attribute embryogenetic pathways to the neurilemmoma and angioma component co-occurrence in the same tumor due to their shared ectomesenchymal origin [3]. In contrast, a genetic understructure has been implicated in other hypotheses where tuberin/hamartin interactions with Rheb1/KREV1RAP1A might play essential roles in the tumorigenesis of these conjoined tumors [3].

Previous studies have shown that these are conjoined lesions having a significant risk of bleeding, particularly when occurring at vestibular locations. It has been hypothesized that tumor cytokines can induce high expression levels of matrix metalloproteinase-2 and -9. This in turn can lead to vascular instability and increased predisposition to thrombosis and bleeding of the aberrant arteries. Subarachnoid hemorrhage can be a serious complication if these tumors occur in the central nervous system (CNS). Further vascular malformations and PNSTs can exhibit confounding and overlapping imaging characteristics. PNSTs are hypointense on T1-weighted imagining and hyperintense on T2-weighted imaging, with the features improving at higher contrast. Hence they can be misinterpreted as vascular malformations; radiologists can be misled into false diagnosing one for the other [14].

## Conclusions

Common tumorigenic molecular pathways might be involved in the conjoined development of schwannoma and hemangiomas. The frequent documentation of these cases implies the inclusion of a separate morphologic entity of 'Angiomatous Variant of Schwannoma' with the existing morphological classification of schwannomas. Vascular malformations and PNSTs can appear similar upon imaging, and PNSTs might be mistaken for vascular malformations. Massive acute/chronic intratumoral hemorrhage and necrosis are common in these tumors, which can clinically and radiologically mimic a malignancy. Hence, awareness of the morphological characteristics of such tumors and documentation of this particular variant may avoid radiological misdiagnoses and surgical mismanagement in the future. 
